# In/Visible Physical Education and the Public Health Agenda of Physical Literacy Development in Hong Kong

**DOI:** 10.3390/ijerph17093304

**Published:** 2020-05-09

**Authors:** Kim-Wai Raymond Sum, Ming-Hui Li, Siu-Ming Choi, Yan Huang, Rui-Si Ma

**Affiliations:** 1 Department of Sports Science and Physical Education, The Chinese University of Hong Kong, Hong Kong, China; venuslmh@link.cuhk.edu.hk (M.-H.L.); choisiuming@link.cuhk.edu.hk (S.-M.C.); Hayleyhy@link.cuhk.edu.hk (Y.H.); penny@link.cuhk.edu.hk (R.-S.M.); 2 School of Physical Education, Jinan University, Guangzhou 510632, Guangdong, China

**Keywords:** physical literacy, physical education, public health, Hong Kong

## Abstract

In this article, we will explore the recent development of physical literacy in the Hong Kong context and how the concept and operation of physical literacy implicitly exist at different levels of the Hong Kong education system. The Physical Education profession will be introduced. The development of physical literacy in terms of research and operationalization in primary, secondary, and tertiary education will then be discussed. We will go on to explore the challenges of extending the impact of physical literacy to the field of public health in Hong Kong. The article will end with a closing remark adopting the Chinese philosophies of Confucianism and Taoism to justify the belief that physical literacy is both implicitly and invisibly rooted in the Hong Kong Chinese culture.

## 1. Background

The use of the concept of physical literacy dates back to 1884 by engineers in the United States Army Corps of Engineers [1]. It was further outlined in the Journal of Health and Physical Education, with the description that public schools should be responsible for physical literacy and mental literacy in 1938, which was the first time the term appeared in educational research [2]. From the philosophical and educational point of view, the terms that we have often adopted describe embodied dimensions including physical activity, strong, fit, physically able, physically educated, etc. However, all of these terms focus on the body as an object under sport, school or manual work contexts, which is prone to the misunderstanding of physical and mental dualism, and the neglect of the embodied capacity of each individual [3]. Consequently, Whitehead [4] opened a debate on the concept of physical literacy based on monism, after which the terminology of physical literacy has once again attracted widespread attention in academia, and has been developed and embraced by researchers worldwide. As appropriate to each individual, a widely adopted physical literacy can be described as the motivation, confidence, physical competence, knowledge and understanding to value and take responsibility for engaging in physical activities for life [5]. The connotation of physical literacy includes physical, cognitive and affective domains, which could be described as a disposition or an attitude acquired by an individual throughout a lifetime. It may be said that physical literacy is relevant to everyone no matter what age and endowment.

Previous research work related to physical literacy has mainly focused on children and adolescents within the school or sport setting since fostering physical literacy for young people is crucial and may influence later participation in life [6]. As early adopters of physical literacy, England, Wales, and Canada have implemented established initiatives and contributed to the development of physical literacy among the younger generation (e.g., Top Sportsability in England, The Foundation Phase in Wales and Passport for Life in Canada) [7]. With the foundation of the International Physical Literacy Association in 2014, physical literacy has become a global concern in the fields of physical education and recreation. Increasing countries and regions (i.e., Hong Kong) have been involved in its promotion.

The term “physical literacy” has rarely been used in either the education system or the physical education curriculum in Hong Kong. Like other countries, literacy has been conceptualized and narrowly defined as the ability to read, write, and use numeracy, and is widely used in subjects such as languages and mathematics in the Hong Kong context. Physical literacy was first introduced to physical education teachers through a continuing professional development program in 2015 which is commissioned by the Education Bureau. Since then, limited research activities in university and operationalizing physical literacy have been developed in university, community, and primary and secondary school settings. Despite the fact that Cairney et al. [8] constructed an evidence-informed conceptual model, the advocacy of physical literacy in the physical education setting has not yet been brought to the interest of its allied fields such as public health in the Hong Kong context. This is in contrast to the attention paid to the impact of physical activity on health and well-being.

In this article, we will explore the recent development of physical literacy in the Hong Kong context and how the concept and operation of physical literacy implicitly exist at different levels of the Hong Kong education system. We will then discuss the challenges inherent in extending the impact of physical literacy to the field of public health.

## 2. The Physical Education Profession and Its Place in the Hong Kong Education System

The education system of Asian countries is ‘examination oriented and it views physical education as a component of play and leisure rather than an intrinsic part of the educational process’ [9] (p. 214). Physical education became one of the eight Key Learning Areas (KLAs) in 2002 [10]. Students are entitled to have a learning experience from physical education throughout their primary and secondary schooling. It is also suggested that 5–8% of the total curriculum time be allocated for the physical education KLA in both primary and secondary schools. Physical education can provide opportunities for teachers to lead students back to a healthy lifestyle through a new health-oriented curriculum, a good teaching and learning atmosphere and an authentic assessment scheme [11]. Studies showed that physical education has been viewed as a minor subject despite the fact that it is regarded as one of the KLAs [12].

The majority of Hong Kong physical education teachers are trained in the Chinese University of Hong Kong (CUHK) and the Education University of Hong Kong (EdUHK). Only a small number are from other local or foreign universities, from which they have usually gained an additional post-graduate diploma majoring in physical education. However, there is no formal course specific to the concept of physical literacy in the physical education teacher educator’s program in these two local universities. For physical education teachers, they are required to fulfill 150 h of continuing professional development in a consecutive three-year period. The Education Bureau of Hong Kong SAR offers varied, year-round programs for physical education teachers both at primary and secondary levels. Physical education teachers are clearly an essential component of the education system. Physical education is important to all students who attend school and is particularly salient for those who are responsible for the well-being of students—physical education teachers [12]. However, there has been considerable debate around the essential mission of these teachers [13,14,15]. Unlike other well-developed education systems in the world, qualified professional physical education teachers are needed to be subject specialists in both primary and secondary education in Hong Kong. Thus, there are no “generalists” in teaching physical education in the education system, as stipulated by the Education Bureau of Hong Kong. Those qualified physical education subject specialists can, at the same time, teach other subjects such as English or Mathematics. In contrast, non-physical education subject specialists are ineligible to teach physical education in primary and secondary schools in Hong Kong. Thus, the roles and responsibilities of a physical education subject specialist are crucial to nurturing students’ lifelong physical activity and motivating students to be active.

The professional roles of a physical education teacher have been specified in the T-standard of the Committee on Professional Development of Teachers and Principals (COTAP) [16]. The T-standard is a unified set of standards for the teaching profession that portrays the professional roles and visions of teachers and principals. The T-standard also supports teachers and principals in planning their professional growth, providing a clear reference and goal for continuing professional development, teacher preparation and development of school leadership [16]. In particular, the T-standard expects the essential roles of physical education teachers to become ‘caring cultivators’, ‘inspirational co-constructors’ and ‘committed role models’ that nurture students to possess whole-person wellness, key competences for adulthood, and change agility for tomorrow [16] (p. 13). The essence of this T-standard approach to students’ development echoes with the goals of physical literacy as being centrally relevant to preadolescent young people, used to identify and nurture talent [17] and, therefore, is considered to be implicitly inherent in the operationalization of physical education in the Hong Kong education system.

## 3. The Development of Physical Literacy in Hong Kong

In Hong Kong, there is no further update on physical literacy in the physical education curriculum guidelines after the introduction through a continuing professional development in 2015. The curriculum and assessment in primary and secondary schools is school-based and does not correspond to an ideal concept of physical literacy. Additionally, researchers have found that less than half of the children and adolescents in Hong Kong met the recommended physical activity level [18]. In the process of encouraging students’ participation in physical activity and the development of physical literacy, the role of physical education teachers is rather important in the school context.

In response to the global exhortation, Sum et al. [19] constructed and validated the Perceived Physical Literacy Instrument (PPLI) among primary and secondary physical education teachers, and also emphasized the importance of physical literacy in directing students’ participation in physical activity. Accordingly, researchers could use the validated PPLI to investigate the self-perception of physical education teachers on physical literacy and whether students influenced towards the same goal. With further validation, this instrument could also be applied to the adolescent population [20]. Choi et al. [21] pointed out a positive relationship between perceived physical literacy and physical activity levels among adolescents in Hong Kong. In addition, Li et al. [22] suggested that a new perspective for coaching the group of student-athletes may be introduced through implementing the concept of physical literacy.

For students in primary school, researchers have translated and adopted the Chinese version of the Canadian Assessment of Physical Literacy-2 (CAPL-2) [23] to measure their actual physical literacy level. Meanwhile, a certification course for training qualified children’s physical literacy assessment leaders was successfully conducted by the Physical Fitness Association of Hong Kong, China, in 2019. A recent finding has reported a significant association between children’s perceived and actual levels of physical literacy through PPLI and CAPL-2 [24]. PPLI was also applied among university students in a Sport Education intervention, followed by the suggestion that the domains of physical literacy should be emphasized in the future [25].

The above description of the development of physical literacy in the Hong Kong context is primarily focused on the school and physical education through a researching approach. There has been no explicit development or promotion of physical literacy in the sport system with reference to the Long-term Athletic Development (LTAD) model [26]. An official revision of curriculum and assessment relating to the concept of physical literacy is guaranteed. However, the development of physical literacy is not only relevant within the school context; it is the result of one’s lifespan [27]. It is worthwhile and, indeed, necessary to develop physical literacy for those in other age groups or people with special needs (e.g., older adults, people with disabilities) so as to contribute to flourishing human life [17].

### 3.1. Primary School Physical Literacy Development

Since it was established, the “Whiteheadian” definition of physical literacy has been supported by more than 1300 physical activity leaders and organizations, including Hong Kong [23]. Meanwhile, the Curriculum Development Council [10] proposed one of the seven learning goals during the “Basic Education” (primary 1–6) period that is to lead a healthy lifestyle and develop an interest in aesthetic and physical activities and an ability to appreciate these activities. Although the exact wording of physical literacy does not exist in the goal, its underpinning concept plays an essential role in acquiring motivation, confidence, and physical competence in respect of participation in physical activity [28]. It has to be said, however, that in Hong Kong, related curriculum policy shows that physical literacy is implied, but undeveloped. This is urgently required [19].

As a multidimensional construct, the measure of physical literacy is especially emphasized through developing its assessments among Hong Kong primary school children, since Dudley et al. [29] assumed that the development of physical literacy, particularly early in life, will influence subsequent physical activity participation and related outcomes across the course of life. As the first valid, reliable, and comprehensive protocol for monitoring actual levels of physical literacy among Canadian children [23], the Canadian Assessment of Physical Literacy-2 (CAPL-2) was adopted to measure actual physical literacy level among Hong Kong primary school children. According to the score distribution pertaining to show children’s physical literacy levels, it was found that nearly all the Hong Kong Chinese children (98.8%) are at the beginning or progressing stage of their physical literacy development [30]. Meanwhile, Li et al. [24] reported a significant association between children’s physical literacy perceptions and their actual levels of physical literacy by using the validated PPLI. They also found gender differences in the daily behavior domain of actual physical literacy measured by the Chinese version of the CAPL-2, with boys scoring significantly higher than girls in daily behavior.

Compared with the children’s actual physical literacy level reported by Canada [23], Greece [31], and African countries [32], the Hong Kong Chinese children’s physical literacy level seems unsatisfactory despite a flattering community environment [33]. Action and efforts of government towards promotion and curriculum policy measures should be taken in order to incorporate the concept of physical literacy officially into physical education, which may play an important role in arousing the awareness of the enhancement of children’s physical literacy.

### 3.2. Secondary School Physical Literacy Development

The development of physical literacy is absent in the Hong Kong secondary school physical education curriculum; rather, it focuses on the learning of motor and sports skills, and the development of physical fitness through physical activities. Teachers should include the learning targets of sport-related values and attitudes, safety knowledge, movement knowledge, and aesthetic sensitivity during the lessons [10]. To cultivate the concept of physical literacy in secondary physical education, a PPLI [19] was further validated in a group of adolescents [20]. Although the instrument is limited in the attributes of confidence, communication, and knowledge, results showed a positive relationship between perceived physical literacy and physical activity levels among Hong Kong adolescents [21].

To construct a more meaningful connotation of the importance of physical literacy of adolescents, advanced research methods and tools, including practical intervention programs and objective measures of physical activity levels by using accelerometers, were recommended. In facilitating the reformation of a physical education program aligned with curriculum towards the attributes of motivation, confidence, physical competence, and knowledge and understanding, Sum et al. [34] proposed a randomized controlled trial of continuing professional development. It was hypothesized that the program could develop teachers’ self-efficacy and knowledge of physical literacy, and then influence students’ learning outcomes, i.e., motivation, confidence, self-report, and objective measure physical activity levels.

Furthermore, the curriculum should promote, expose and boost the opportunities of students to participate in co-curricular physical activities organized by sports associations [10]. Given more freedom without school restrictions, students could choose their recreational physical activities and experience the physical literacy journey with coaches and peers in sports clubs and community centers [21]. Meanwhile, Li et al. [22] found the mediation effect of leadership behavior between perceived physical literacy and coaching efficacy of student-athletes. Coaches, who are also stakeholders, could also organize development programs embedding the concept of physical literacy to enhance their coaching.

Considering the research studies conducted around the world and in the context of Hong Kong recently, the incorporation of physical education curriculum and physical literacy is recommended. Although the skill-oriented curriculum guidelines suggested by the Education Bureau omitted the concept of physical literacy, it mentioned the knowledge aspects and the importance of lifelong physical activities. The prospective curriculum should invigorate the planning of game-based physical education lessons in promoting students’ physical literacy, especially the affective domain of motivation and confidence. It would also be valuable to determine whether recreational physical activity programs could provide the maximum effect on physical literacy and participation in physical activities for adolescents.

### 3.3. Tertiary Level Physical Literacy Development

Following on the suggestion of expanding the use of the PPLI, this could further apply to university students because they are encountering the transition from compulsory physical education to more self-initiated physical activities behavior [20]. In the tertiary physical education of Hong Kong, two universities have organized teacher education programs, and most of the universities have organized typical physical education or physical activity courses, but only one of them has set one of the graduation requirements as completing two physical education courses within the normative study period.

In the context of tertiary physical education, the sole specific curriculum guideline is based on the autonomous decision by the individual university. The only university which provides the required physical education courses has been organizing a series of continuing professional development programs for their lecturers to develop and apply Sport Education in promoting physical literacy. After a 15-week Sport Education intervention, findings showed a greater increase in daily self-reported physical activity levels in the Sport Education group than the control group during the follow-up phase given the improvement in the affective and social domains of physical literacy across both groups [25]. Future studies are needed to address the domains of physical literacy and to understand the practical perspective of physical education professionals providing such pedagogical methods in a pragmatic approach of mixed-method study. Since there is only one university in Hong Kong providing such an opportunity for their students to experience physical education, the remaining universities could also provide a unique environment to promote the essentials of physical literacy and a positive attitude towards physical activity.

In synthesizing with the quality physical education (QPE) guidelines [35], teacher education institutes should not only emphasize physical literacy in professional development but also within the group of pre-service physical education teachers. By revealing the positive relationship between perceived physical literacy and teaching efficacy, teacher educators should continue to promote the importance of physical literacy, encourage pre-service physical education teachers to reflect on their past physical activity experiences, and apply related methods during supervised teaching practice and professional practice [36]. Future study directions could also align with the pragmatic approach of charting physical literacy [37,38] in order to reaffirm the significance of this relationship. This can be carried out by using qualitative methods to examine teachers’ reasons for committing to the field of physical education and their experience of the teacher education program, as well as the physical activities of pre-service physical education teachers.

As physical education at the tertiary level encounters the transition from a compulsory situation, which is policy-driven, to more self-oriented participation in physical activities, the autonomous motivation to participate in sport and physical activity seems far more essential to cultivate. Indeed, the pragmatic approach of the physical literacy-enriched experience and understanding would be more effective. In addition, generating a participatory climate of more workshops, programs, and research projects would be very useful for attracting the involvement of the young adult generation.

As only one university in Hong Kong provides such an opportunity for students to experience physical literacy-oriented physical education courses currently, other universities could acquire beneficial information from these projects to help more students to promote physical literacy and a positive attitude towards physical activity in their upcoming life journey as adults.

## 4. In/Visible Public Health Agenda

Whether or not physical education can be replaced by another name—physical literacy—may cause confusion among physical educators. The major concern is the apparent loss of increasing physical activity and physical fitness as an outcome in physical education [39]. In fact, physical education was dominated by physicians who specialized in health and exercise in the early 1900s [14], showing that physical activity and regular exercise were more central to public health. From the historical perspective, physical activity has always been a hot topic in the field of public health related to preventive medicine, chronic diseases and so on. The rekindling of interest in the role of physical activity and even physical literacy played in health came along with the rise of lifestyle physically active pursuits like aerobics, cycling, and running. Despite the fact that ongoing research proves the importance of physical activity in supporting health outcomes [40], and that physical literacy is associated with physical activity [21], physical literacy as a determinant in the field of public health is still not an agenda item to date in Hong Kong.

The concept of physical literacy should be spreading to those who are involved in working with children in the early years, participants with disabilities, the medical profession, and those who care for the elderly [17]. This is also justified since there is potential for the concept of physical literacy to be developed in the field of public health. In the context of physical education, physical literacy has been promoted to teachers and government officials in previous continuing professional development programs. Some additional support programs including talks, seminars, and workshops could be organized for parents through the parent–teacher association in each school. Parents, K-12 teachers in each content area and principals could then construct a platform through these activities and become more knowledgeable on the importance of developing students’ academic and physical literacy simultaneously. This could also prevent the marginalization of physical education which may reduce time and resource allocation in a school. Since Cairney et al. [8] proposed a ‘physical literacy, physical activity and health toward an evidence-informed conceptual model’, physical literacy has more role to play in promoting positive health behaviors than physical literacy positioned as one of the health determinants. However, there has been almost no attention to the positioning of physical literacy in the field of public health in Hong Kong.

With regard to the physical literacy promotion and policy, there are seven regions including England, Wales, Canada (Montreal), Canada (Toronto), the United States, New Zealand, and Australia, that have their definitions of physical literacy, which means physical literacy’s important message is being disseminated to the public. In line with this, Hong Kong could also collaborate with other Asian countries and the Greater China Region including China, Taiwan and Macau to develop a strategic plan, intervention and assessment of physical literacy in the physical education and public health context. However, studies conducted for Hong Kong children and adolescents revealed a low level of objectively measured physical literacy, consistent with the previous report on children and youth’s physical activity participation synthesized through the data reviewed from the past ten years [33]. The findings greatly recommended the incorporation of physical education curriculum and physical literacy into the public health policy measure officially. To stimulate empirical research, there is an ongoing small-scale research “Charting and developing physical literacy for older baby boomers” in the aspects of physical competency, motivation, knowledge and understanding, and social domains. The project aims to implement the mixed habit-based invention for developing a physical literacy journey among older baby boomers (65–75 age) in Hong Kong. There is still an urgent need to develop physical literacy for this group so as to contribute to healthy aging in Hong Kong.

Along this line, Blair [41] argued that evidence shows physical inactivity is one of the most important public health problems of the 21st century. Physical literacy at this point could provide policymakers and public health researchers an avenue or alternative view to exploring the impact that physical literacy could make to Hong Kong and every corner of the globe. Topping et al. [42] noted that the Scottish Government was trying to develop healthy school communities. The curriculum that ‘promotes learning through health and well-being in ways that develop confidence and understanding in order to develop mental, emotional, social and physical well-being’ (p. 182) is currently underway in Scotland. This approach to public health is well-suited to the philosophy underpinning physical literacy and to which the field of public health should make reference in the Hong Kong context.

## 5. Concluding Remark

In this article, the last remark is that Hong Kong is rooted deeply and firmly in Chinese philosophy [43,44]. Regardless of the fact that there is no formal curriculum and policy in promoting physical literacy, the concept of physical literacy has implicitly been nurtured in the Hong Kong education system with our Confucianism and Taoism perspectives. A Confucian motto related to being physically literate such as ‘Equip oneself; A well-managed family; Run the country well; Bring peace to the world’ [45] has been commonly taught and penetrates the school systems in Hong Kong. The Taoist approach of “Xiuyang” (a holistic approach of “literate”) is also a concept for health and spiritual preservation throughout the Chinese culture including Hong Kong [46]. Currently, physical literacy was mainly promoted through research-based projects, further revision of its status in the curriculum and assessment is warranted. Still, the Chinese philosophy, by definition, is by some means affiliated with the monism underpinnings of physical literacy. Therefore, physical literacy has implicitly or invisibly been understood by the Hong Kong people. To seek “harmony in diversity”, the role of the Chinese philosophy is to balance between idealist and pragmatic perspectives of physical literacy, particularly, whether promote this concept from the philosophical view, from the sport and fitness approach, or both. Nevertheless, pursuing a monistic viewpoint could align with both Chinese philosophies and previous scientific findings. To benchmark with other well-developed physical literacy countries or regions, it is hoped that this article supports policymakers, educators, and researchers in Hong Kong and different regions of the world where it is a call for insightful, informed, situational, and contextual specific judgements for promoting physical literacy.
